# Effects of guanidinoacetic acid on *in vitro* rumen fermentation and microflora structure and predicted gene function

**DOI:** 10.3389/fmicb.2023.1285466

**Published:** 2024-01-09

**Authors:** Chenyang Dong, Manlin Wei, Ji Ju, Liu Du, Runze Zhang, Ming Xiao, Yongjie Zheng, Hailin Bao, Meili Bao

**Affiliations:** ^1^College of Animal Science and Technology, Inner Mongolia Minzu University, Tongliao, China; ^2^Horqin Left Wing Rear Banner Ethnic Vocational and Technical School, Tongliao, China

**Keywords:** gas production, fermentation parameters, rumen microorganisms, gene function, metabolic pathway 1

## Abstract

The fermentation substrate was supplemented with 0% guanidinoacetic acid (GAA) (control group, CON), 0.2% GAA (GAA02), 0.4% GAA (GAA04), 0.6% GAA (GAA06) and 0.8% GAA (GAA08) for 48 h of *in vitro* fermentation. Gas production was recorded at 2, 4, 6, 8, 12, 24, 36, and 48 h of fermentation. The gas was collected, and the proportions (%, v/v) of H_2_, CH_4_ and CO_2_ were determined. The rumen fermentation parameters, including pH, ammonia nitrogen (NH_3_-N), microbial protein (MCP) and volatile fatty acids (VFAs), were also determined. Furthermore, the bacterial community structure was analyzed through 16S rRNA high-throughput sequencing. The gene functions were predicted using PICRUSt1 according to the Kyoto Encyclopedia of Genes and Genomes (KEGG). The results showed that with the increase in GAA supplementation levels, the MCP and the concentration of rumen propionate were significantly increased, while the concentration of isovalerate was significantly decreased (*p* < 0.05). The results of microbial diversity and composition showed that the Shannon index was significantly decreased by supplementation with GAA at different levels (*p* < 0.05), but the relative abundance of *norank_f_F082* and *Papillibacter* in the GAA06 group was significantly increased (*p* < 0.05). Especially in group GAA08, the relative abundances of Bacteroidota, *Prevotella* and *Prevotellaceae_UCG-001* were significantly increased (*p* < 0.05). The results of gene function prediction showed that the relative abundances of the functions of flagellar assembly, bacterial chemotaxis, plant–pathogen interaction, mismatch repair and nucleotide excision repair were significantly decreased (*p* < 0.05), but the relative abundances of bile secretion and protein digestion and absorption were significantly increased (*p* < 0.05). In conclusion, supplementation with 0.8% GAA enhanced *in vitro* rumen fermentation parameters, increased the relative abundance of *Prevotella* and *Prevotellaceae_UCG-001* in the rumen, and increased the metabolic pathways of bile secretion and protein digestion and absorption.

## 1 Introduction

Creatine is phosphorylated to produce phosphocreatine, which can provide energy for cell activities. Creatine produced inside the body of animals can only meet 50–70% of the body’s needs, and the remaining amount should be supplemented by exogenous creatine (Ostojic, 2017). Creatine has a high production cost, poor stability, and a low utilization rate of additional creatine in animals (Chamruspollert et al., 2002). Guanidinoacetic acid (GAA), the direct precursor of creatine biosynthesis in animals, has stable chemical properties and high biological value and is suitable for use as a feed additive (Baker, 2009). Currently, GAA, as a nutritional feed additive, has been approved for use in animals by the European Food Safety Authority (Poaa, 2016) and the Chinese Ministry of Agriculture (MOA Ministry of Agriculture (PRC), 2014). Supplementation of GAA in poultry diets was proven to enhance the feed conversion rate and muscle quality (Lemme et al., 2007; Khalil et al., 2021), as well as liver antioxidant capacity (Zhao et al., 2021) and muscle energy metabolism (Michiels et al., 2012). In addition, GAA supplementation can significantly increase the average daily feed intake, feed conversion and lean meat percentage of pigs (Jayaraman et al., 2018) and improve carcass traits and meat quality by reducing the mandibular fat index and changing muscle fiber characteristics (Zhu et al., 2020). Liu et al. (2021) reported that dietary supplementation with 0.6 g/kg GAA significantly increased the body weight and average daily gain of Angus bulls.

In fact, the function of the rumen is very important for nutrition. Rumen microbes can degrade protein in feed to provide amino acids as a nitrogen source for the body (Sari et al., 2022) and digest carbohydrates to produce VFAs as an energy source (Wilk et al., 2021). Research on GAA has mainly focused on animal growth, meat quality, and antioxidant properties of poultry and pigs (Lemme et al., 2007; Jayaraman et al., 2018; Khalil et al., 2021; Zhao et al., 2021). However, only a few studies have investigated the effect of adding GAA on ruminants, and the effect of GAA on rumen fermentation and rumen microflora is unclear. Therefore, an *in vitro* experiment was carried out to study the effects of different levels of GAA on rumen gas production, gas composition and fermentation parameters, as well as the structure and gene function of rumen microflora through 16S rRNA high-throughput sequencing technology, to provide a reference for the use of GAA in ruminant feed.

## 2 Materials and methods

The GAA used in the experiment was provided by Beijing Gendone Biotechnology Co., LTD. GAA is a white powder with 98% purity. All procedures in this study were approved by the Laboratory Animal Ethics Committee of Inner Mongolia Minzu University (protocol number: 2018090516001).

### 2.1 Experimental animals and design

Four healthy Simmental hybrid female cattle (500 kg ± 30 kg) equipped with permanent rumen fistulas were selected as rumen fluid donors, and rumen fluid was collected from the fistulas for *in vitro* fermentation experiments. The basal diet of cattle was formulated according to the NRC (2016), and cattle were fed twice a day and allowed to drink freely. The ingredients and nutrients of the diet for the cattle are shown in Table 1. The experiment adopted a single-factor experimental design, with 0% (control group, CON), 0.2% (GAA02), 0.4% (GAA04), 0.6% (GAA06), and 0.8% (GAA08) GAA supplemented to the fermentation substrate (same as the diet of fistula cattle), with three replicates in each group.

**Table 1:** Composition and nutrient composition of the diet for cattle with fistulas (dry matter basis).

Ingredients	Content (%)	Nutrients^2^	Content (%)
Corn stalk	60.00	NE/MJ·kg^−1^	6.84
Corn	16.20	Dry Matter, DM	89.12
Soybean meal	12.80	Crude protein, CP	13.19
Corn germ meal	5.20	Ether extract, EE	3.14
Distillers dried grains with soluble	1.60	Neutral detergent fiber, NDF	47.17
Limestone	0.96	Acid detergent fiber, ADF	34.87
Sodium bicarbonate	0.72		
Calcium hydro phosphate	0.84		
Salt	0.68		
Premix^1^	1.00		
Total	100.00		

1. Premix provided per kg of diet: VA 320000 IU, VD 8000 IU, VE 2200 IU, Cu 710 mg, Fe 900 mg, Mn 900 mg, Zn 1,400 mg, Se 60 mg, I 25 mg, and Co 35 mg. 2. The comprehensive net energy is the calculated value, and the nutrient level is the measured value.

### 2.2 *In vitro* fermentation experiment

The *in vitro* experiment was carried out according to the method of Menke et al. (1979). Approximately 500 mg (dry matter basis) of fermentation substrate (same as the diet of fistula cattle) was weighed and transferred into a glass syringe with a volume of 100 mL, which was calibrated (model HFT000025, Häberle, Germany). The rumen fluid was collected from the 4 fistulae cattle before the morning feeding, filtered with four layers of gauze, mixed into a preheated water bath at 39°C and saturated with CO_2_. Approximately 50 mL of culture solution was added to a glass syringe containing the substrate and preheated to 39°C, and the initial volume of each glass syringe was recorded for calibration. Afterwards, the glass syringe was sealed and placed in a water bath oscillator at a constant temperature of 39°C and shaken at a speed of 120 r/min. The volume of the gas in the glass syringe was read and recorded at 2, 4, 6, 8, 12, 16, 20, 24, 36, and 48 h. The gas in each glass syringe was collected into a 200 mL aluminum foil gas sampling bag (Shanghai Huibin Instrument Co., Ltd., Shanghai, China).

According to the model proposed by Ørskov and Mcdonald (1979), SAS 8.0 software was used for analysis, and dynamic fermentation parameters were calculated:


$$\mathrm{GP}=B\;\left(1-e^{-\mathrm{Ct}}\right)$$

In the formula, GP is the cumulative gas production of the sample at time point t (mL); B is the theoretical maximum gas production of the sample (mL); C is the gas production rate of the sample (mL·h^−1^); and T is the cultivation time (h).

### 2.3 Sample collection

After 48 h of *in vitro* fermentation, the glass syringe was quickly removed from the water bath oscillator and placed in an ice bath to terminate the fermentation. All fermentation fluid was collected in a 50 mL centrifuge tube, and the pH was immediately measured. Then, the fermentation fluid was frozen and stored in a − 20°C refrigerator for ammonia nitrogen (NH_3_-N), microbial protein (MCP), and volatile fatty acid (VFA) concentration determinations. A total of 1.5 mL fermentation fluid was collected into a cryotube and kept at −80°C for microbial flora determination of the V3-V4 area by 16S rRNA sequencing technology.

### 2.4 Determination of dietary indices

The diet sample was continuously dried in a drying oven at 65°C for 48 h to make an air-dried sample, and the DM content was then determined by drying at 105°C to constant weight (Ju et al., 2023). The crude protein (CP) and ether extract (EE) contents were determined using the standard Association of Official Analytical Chemists procedures (AOAC, 1990). The neutral detergent fiber (NDF) and acid detergent fiber (ADF) contents were quantified using the method described by Van Soest et al. (1991).

### 2.5 Determination of the rumen fermentation parameters

The proportions (%, v/v) of H_2_, CH_4_ and CO_2_ in the rumen gas were determined by gas chromatography (TP-2060 T, Beijing Analytical Instrument Co., Ltd.). Instrument conditions: TCD detector, column model TDX-01, 1 m × 3 mm × 2 mm, column temperature 120°C, detector temperature 150°C, injection port temperature 150°C, carrier gas argon, carrier gas flow rate 50 mm/min. The standard gas consisted of 24.80% CH_4_, 65.10% CO_2_, 2.01% H_2_, 3.00% O_2_ and 5.00% N_2_, and the detection volume was 100 μL. Rumen pH was determined using a portable pH meter (SX-620). The concentrations of rumen NH_3_-N, MCP and VFA were determined according to the methods of Broderick and Kang (1980), Makkar et al. (1982) and Wu et al. (2013), respectively.

### 2.6 DNA extraction and PCR amplification

Total microbial genomic DNA was extracted from rumen fluid samples using the E.Z.N.A.® soil DNA Kit (Omega Biotek, Norcross, GA, USA) according to the manufacturer’s instructions. The quality and concentration of DNA were determined by 1.0% agarose gel electrophoresis and a NanoDrop® ND-2000 spectrophotometer (Thermo Scientific Inc., USA) and kept at −80°C prior to further use. The hypervariable region V3-V4 of the bacterial 16S rRNA gene was amplified with primer pairs 338F (5′-ACTCCTACGGGAGGCAGCAG-3′) and 806R (5′-GGACTACHVGGGTWTCTAAT-3′) by an ABI GeneAmp® 9,700 PCR thermocycler (ABI, CA, USA) (Liu et al., 2016).

### 2.7 Illumina MiSeq sequencing and data processing

Purified amplicons were pooled in equimolar amounts and paired-end sequenced on an Illumina MiSeq PE300 platform (Illumina, San Diego, USA) according to the standard protocols by Majorbio Bio-Pharm Technology Co. Ltd. (Shanghai, China). Raw FASTQ files were demultiplexed using an in-house Perl script and then quality-filtered by fastp version 0.19.6 (Chen et al., 2018) and merged by FLASH version 1.2.7 (Magoč and Salzberg, 2011). Then, the optimized sequences were clustered into operational taxonomic units (OTUs) using UPARSE 7.1 with a 97% sequence similarity level (Stackebrandt and Goebel, 1994; Edgar, 2013). The taxonomy of each OTU representative sequence was analyzed by RDP Classifier version 2.2 (Wang et al., 2007) against the 16S rRNA gene database (Silva v138). Based on the OTU information, rarefaction curves, Venn diagrams and alpha diversity indices, including observed OTUs, were calculated with Mothur v1.30.1 (Schloss et al., 2009; Segata et al., 2011). Co-occurrence networks were constructed to explore the internal community relationships across the samples (Barberan et al., 2012). Metagenomic function was predicted by PICRUSt1 based on representative OTU sequences. GraphPad Prism (version 9, IBM, Armonk, NY, United States) was used to map the metabolic pathway results of KEGG. The raw sequencing data of this study are available in the NCBI SRA database with the BioProject ID of PRJNA1011887.

### 2.8 Statistical analysis

The experimental data and the dynamic fermentation parameters were assessed by the one-way ANOVA procedure of John’s Macintosh Project version 13 software (SAS Institute, Japan), and multiple comparisons were made using Duncan’s test. Data are expressed as the mean and standard error (SEM). *P* < 0.05 was considered statistically significant, and 0.05 < *p* < 0.10 was considered to indicate a significant difference in trend.

## 3 Results

### 3.1 Effect of guanidinoacetic acid on *in vitro* gas production and gas parameters

**Figure 1:**
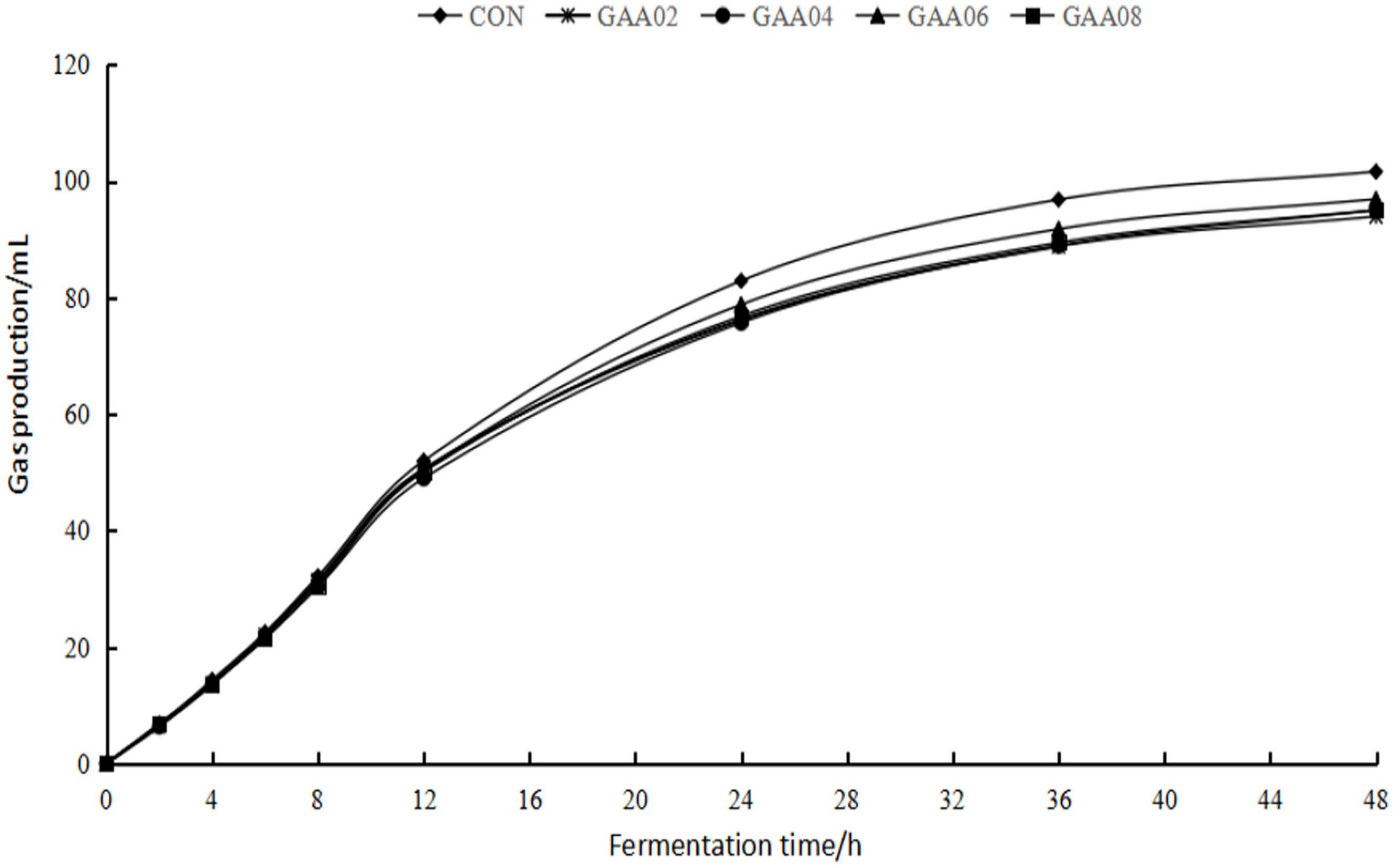
*In vitro* gas production plots from 0 ~ 48 h.

As shown in Figure 1, gas production in the GAA groups decreased to varying degrees compared with that in the CON group from 12 h to 48 h, but there was no significant difference in total gas production at 48 h (GP_48 h_) between all groups (*p* > 0.05) (Table 2). After model prediction based on actual gas production, the theoretical maximum gas production (B) and gas production rate (C) of each group were obtained, which showed no significant difference between all groups (*p* > 0.05). The proportions of H_2_, CH_4_ and CO_2_ in the GAA groups were not significantly different (*p* > 0.05).

**Table 2:** Effects of GAA on rumen fermentation gas parameters.

Items	CON	GAA02	GAA04	GAA06	GAA08	SEM	*p* value
GP_48 h_ /(mL)	100.70	92.85	97.32	97.13	95.07	2.142	0.196
B/(mL)	118.23	107.14	116.01	114.34	110.67	3.514	0.256
C/mL·h^−1^	0.04	0.04	0.04	0.04	0.05	0.001	0.276
H_2_ /%	0.02	0.02	0.02	0.02	0.02	0.002	0.580
CH_4_ /%	14.58	14.75	14.19	14.75	14.93	0.387	0.715
CO_2_ /%	77.78	80.73	78.57	79.07	79.38	1.616	0.768

GP, gas production; B, theoretical maximum gas production; C, gas production rate. SEM, standard error of the mean.

### 3.2 Effect of guanidinoacetic acid on rumen fermentation parameters

According to Table 3, after 48 h of *in vitro* fermentation, the MCP content in the GAA group was significantly higher than that in the CON group (*p* < 0.05), with the GAA08 group having the highest MCP content (*p* = 0.006). The concentration of propionate significantly increased with increasing GAA addition level compared with that in the CON group (*p* < 0.05), while the concentration of isovalerate significantly decreased (*p* < 0.05). There was no significant change in acetate, but the ratio of acetate to propionate (A/P) showed a decreasing trend due to the increase in propionate (*p* = 0.055). Moreover, there were no significant differences in pH, NH_3_-N, butyrate, isobutyrate, valerate, or total volatile fatty acid (TVFA) concentrations between the groups (*p* > 0.05).

**Table 3:** Effects of GAA on rumen fermentation parameters.

Items	CON	GAA02	GAA04	GAA06	GAA08	SEM	*p* value
pH	6.11	6.09	6.13	6.12	6.12	0.033	0.877
NH_3_-N/(mg·dL^−1^)	31.99	33.07	32.90	32.06	35.31	0.940	0.164
MCP/(mg·dL^−1^)	15.35^b^	24.35^a^	22.32^a^	26.14^a^	26.85^a^	1.742	0.006
TVFA/(mmol·L^−1^)	47.34	46.35	46.69	48.04	46.77	2.275	0.985
Acetate/%	66.71	67.06	67.17	67.39	66.76	0.192	0.141
Propionate/%	18.00^c^	18.25^b^	18.42^b^	18.35^b^	18.60^a^	0.106	0.026
Isobutyrate/%	1.07	1.07	1.13	1.10	1.14	0.033	0.510
Butyrate/%	11.65	11.48	11.39	11.52	11.85	0.162	0.373
Isovalerate/%	1.56^a^	1.20^b^	1.00^b^	0.74^c^	0.70^c^	0.147	0.011
Valerate/%	1.01	0.93	0.90	0.91	0.96	0.038	0.302
A/P	3.71	3.68	3.65	3.67	3.59	0.024	0.055

NH_3_-N, ammonia-N; MCP, microbial protein; TVFA, total volatile fatty acids; A/P, acetate/propionate; SEM, standard error of the mean. a, b, c in the same row, there is a statistically significant difference (*p* < 0.05).

### 3.3 Effects of guanidinoacetic acid on rumen bacterial diversity and microbial community structure

#### 3.3.1 Analysis of the rumen bacterial diversity

**Figure 2:**
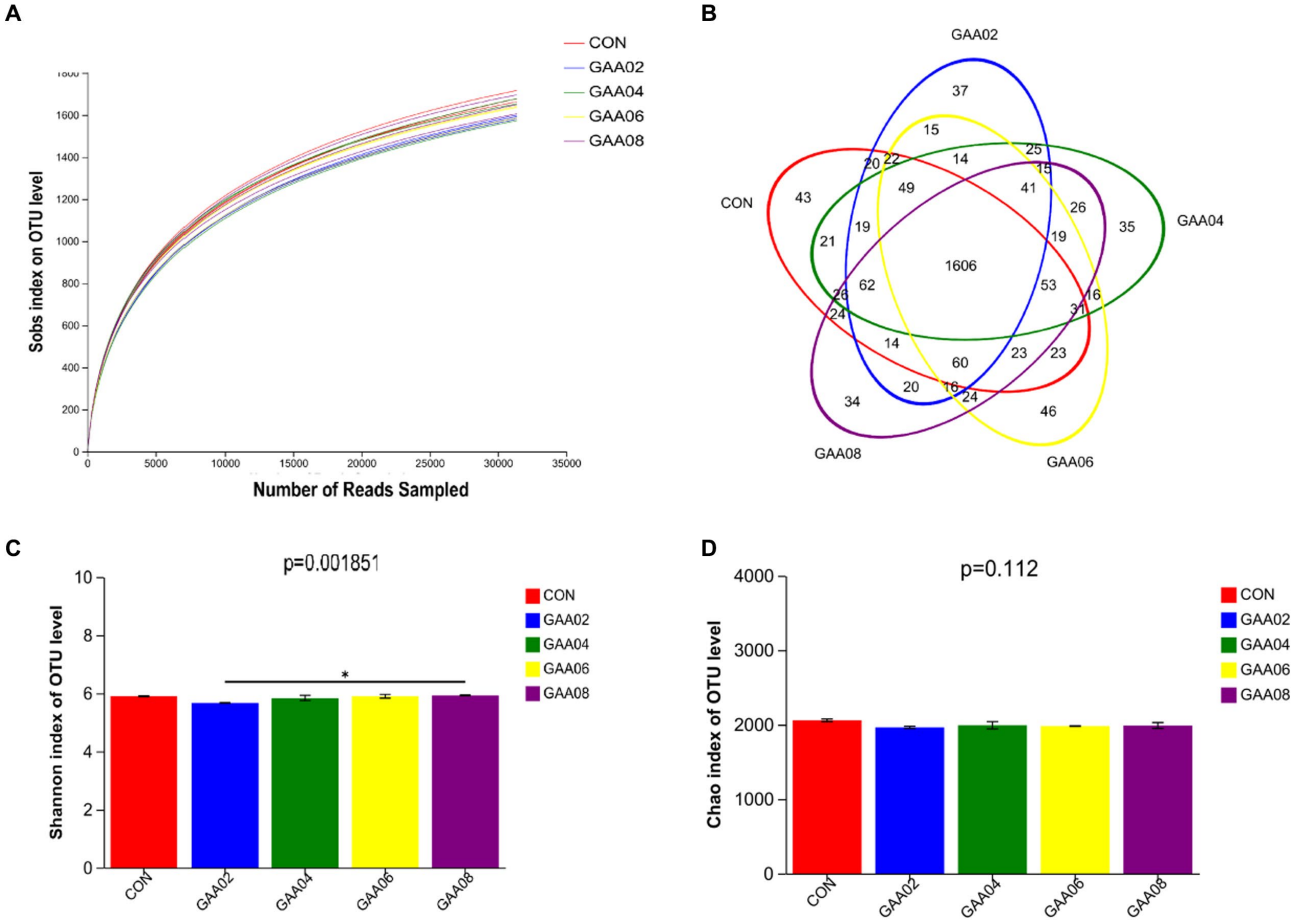
Dilution curve analysis **(A)**, operational taxonomic unit (OTU) Venn analysis **(B)**, Shannon index bar graph **(C)** and Chao index bar graph **(D)**. CON, GAA 02, GAA 04, GAA 06, and GAA08 represent the control group (0%), 0.2, 0.4, 0.6, and 0.8% groups, respectively. *There is a statistically significant difference (*p* < 0.05).

Three rumen fluid samples from each group were filtered by high-throughput sequencing to obtain 470,985 effective sequences. As shown in Figure 2A, the growth rate of the dilution curve slowed, indicating that the amount of sequencing data obtained thus far was sufficient to reflect the diversity of most species in the test samples. Based on the 97% similarity level, effective sequences were OTU clustered (Figure 2B), and a total of 2,479 OTUs were obtained. The number of OTUs in CON group was 2096, GAA02 group was 2035, GAA04 group was 2058, GAA06 group was 2058, and GAA08 group was 2063. The total number of OTUs in the five groups was 1,606, among which the number of unique OTUs was 43, 37, 35, 46 and 34, accounting for 2.05, 1.82, 1.70, 2.23, and 1.65%, respectively. According to the above data, there were differences in the composition of rumen microflora after GAA addition. On the other hand, compared with the CON group, the Shannon index was significantly reduced by supplementation with GAA at different levels (*p* < 0.05), but the Chao index had no significant change (Figures 2C,D), indicating that supplementation with GAA at different levels significantly impacted rumen microbial diversity.

#### 3.3.2 Structural difference analysis of the rumen microflora composition

After taxonomic annotation of species for each sample group, there were 7 phyla with relative abundances greater than 1% (Table 4). They were Firmicutes, Bacteroidota, Actinobacteriota, Spirochaetota, Proteobacteria, Desulfobacterota and Verrucomicrobiota. Among them, Firmicutes and Bacteroidota were the dominant bacteria, and the total relative abundance of Firmicutes and Bacteroidota accounted for more than 91% in each group of bacteria. Compared with the CON group, the relative abundance of Firmicutes first increased and then decreased with increasing GAA addition (*p* = 0.052), while the relative abundance of Bacteroidota first decreased and then increased. The other phyla with relative abundances greater than 1% did not show significant differences between all groups (*p* > 0.05).

**Table 4:** Effects of GAA on the relative abundance of rumen microflora at the phylum level (%).

Items	CON	GAA02	GAA04	GAA06	GAA08	SEM	*p* value
Firmicutes	53.31	59.89	56.86	57.79	47.29	2.671	0.052
Bacteroidota	38.00^b^	31.47^c^	35.76^c^	36.00^c^	44.10^a^	2.203	0.027
Actinobacteriota	2.03	1.90	1.79	1.63	1.66	0.321	0.893
Spirochaetota	1.52	0.88	1.25	1.10	2.89	0.047	0.083
Proteobacteria	1.75	2.63	1.01	0.56	0.93	1.166	0.738
Desulfobacterota	0.83	1.15	0.92	0.93	0.92	0.135	0.584
Verrucomicrobiota	1.17	0.81	1.06	0.79	0.88	0.159	0.405

SEM, standard error of the mean; a, b, c in the same row, there is a statistically significant difference (*p* < 0.05).

A total of 329 genera were obtained after taxonomic annotation of the species. Table 5 shows the top 15 genera in relative abundance. *Rikenellaceae_RC9_gut_group* was the dominant bacteria in the rumen, and its relative abundance accounted for more than 15% of all groups of bacteria. Compared with those in the CON group, the relative abundances of *norank_f__F082* (*p* = 0.023) and *Papillibacter* (*p* = 0.014) in the GAA06 group were significantly increased. The relative abundances of *Prevotella* (*p* = 0.026) and *Prevotellaceae_UCG-001* (*p* = 0.046) in the GAA08 group were significantly increased. For *Christensenellaceae_R-7_group*, *norank_f__UCG-011*, *Succiniclasticum*, *NK4A214_group*, *Lachnospiraceae_NK3A20_group*, *Saccharofermentans*, *norank_f__Eubacterium_coprostanoligenes_group*, *Family_XIII_AD3011_group*, *Acetitomaculum* and *norank_f__Muribaculaceae*, there were no significant differences in the relative abundance of the nine bacteria in all groups (*p* > 0.05).

**Table 5:** Effects of GAA on the relative abundance of rumen microflora at the genus level (%).

Items	CON	GAA02	GAA04	GAA06	GAA08	SEM	*p* value
*Rikenellaceae_RC9_gut_group*	18.01	15.91	16.59	18.69	15.64	1.037	0.239
*Prevotella*	7.14^b^	5.79^b^	7.73^b^	4.17^b^	13.13^a^	1.616	0.026
Christensenellaceae_R-7_group	5.18	6.43	5.55	6.69	4.66	0.567	0.138
*norank_f__UCG-011*	5.62	7.54	6.17	3.81	4.84	1.267	0.359
*Succiniclasticum*	6.28	5.81	6.17	3.63	4.70	1.273	0.558
*norank_f__F082*	5.32^b^	4.13^d^	4.23^c^	5.75^a^	4.08^d^	0.363	0.023
*NK4A214_group*	3.55	3.91	3.65	4.27	3.30	0.249	0.144
*Lachnospiraceae_NK3A20_group*	2.65	3.06	2.85	3.24	2.42	0.303	0.388
*Papillibacter*	2.43^b^	2.08^b^	2.07^b^	3.03^a^	2.13^b^	0.176	0.014
*Saccharofermentans*	2.11	2.07	2.22	2.38	2.05	0.103	0.203
*norank_f_Eubacterium_coprostanoligenes_group*	1.94	1.95	1.95	1.77	1.63	0.248	2.847
*Family_XIII_AD3011_group*	1.75	2.05	1.78	1.30	1.38	0.238	0.237
*Acetitomaculum*	1.42	1.56	1.56	1.69	1.34	0.137	0.447
*Prevotellaceae_UCG-001*	1.17^b^	0.87^b^	1.36^b^	1.15^b^	2.39^a^	0.310	0.046
*norank_f__Muribaculaceae*	1.19	1.19	1.10	2.24	0.91	0.423	0.266

SEM, standard error of the mean; a, b, c, d in the same row, there is a statistically significant difference (*p* < 0.05). The intermediate rank in the taxonomic lineage has no scientific name labeled norank.

#### 3.3.3 Correlation of the rumen fermentation parameters with the microbial community

**Figure 3:**
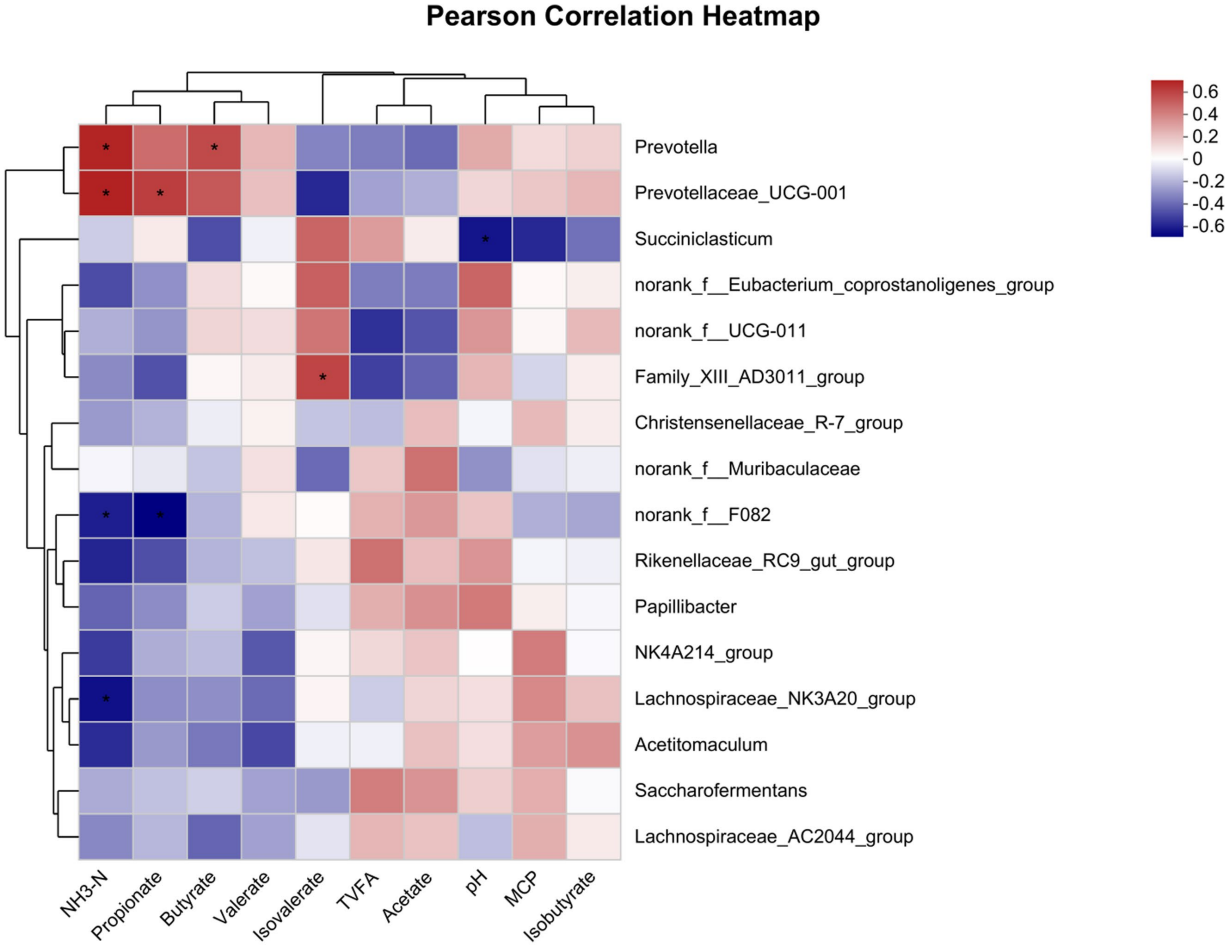
Correlation of the rumen fermentation parameters with the microbial community. The R values are shown in different colors in the graph, and the legend on the right is the color interval of the different R values. *p ≤ 0.05.

The correlation between relative abundance and fermentation parameters of the top 16 bacterial genera (Figure 3). NH_3_-N was negatively associated with the relative abundance of *norank_f__F082* and *Lachnospiraceae_NK3A20_group* (*p* < 0.05), and it was also positively correlated with the relative abundance of *Prevotella* and *Prevotellaceae_UCG-001* (*p* < 0.05). Propionate was negatively associated with the relative abundance of *norank_f__F082* (*p* < 0.05), and it was also positively correlated with the relative abundance of *Prevotellaceae_UCG-001* (*p* < 0.05). The relative abundance of *Family_XIII_AD3011_group* correlated positively with isovalerate (*p* < 0.05), and the relative abundance of *Prevotella* correlated positively with butyrate (*p* < 0.05). The relative abundance of *Succiniclasticum* correlated negatively with pH (*p* < 0.05).

#### 3.3.4 Network plot of the rumen microbial species correlation

**Figure 4:**
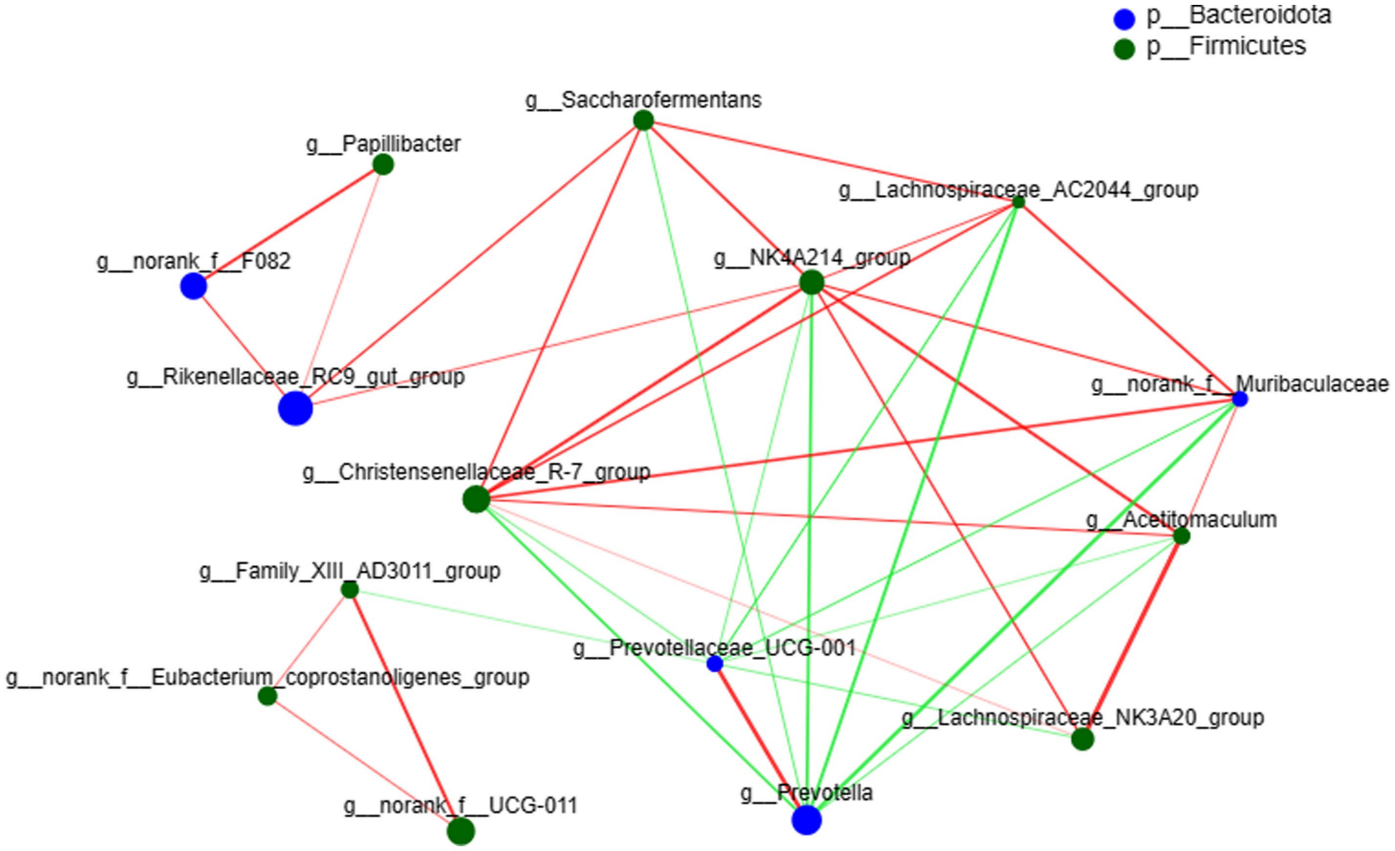
Network plot of species correlation at the genus level. The size of the nodes in the figure represents species abundance, the red line represents a positive correlation, and the green line represents a negative correlation. The thicker the line, the higher the correlation between species; the more lines there are, the more closely related the species is to other species.

The species correlation network diagram mostly reflects the species correlation at various classification levels under certain environmental conditions and calculates the Spearman rank and other correlation coefficients between species. Correlation network analysis was performed on the top 15 species with total abundance at the genus level (Figure 4), where 10 genera belong to Firmicutes and 5 genera belong to Bacteroidota. *Prevotella* was positively correlated with *Prevotellaceae_UCG-001*, and both were negatively correlated with other bacteria. The number of bacterial nodes exceeded 7, including *NK4A214_group*, *Prevotella*, *Prevotellaceae_UCG-001* and *Christensenellaceae_R-7_group*.

### 3.4 Effect of guanidinoacetic acid on gene function prediction in rumen bacteria

**Figure 5:**
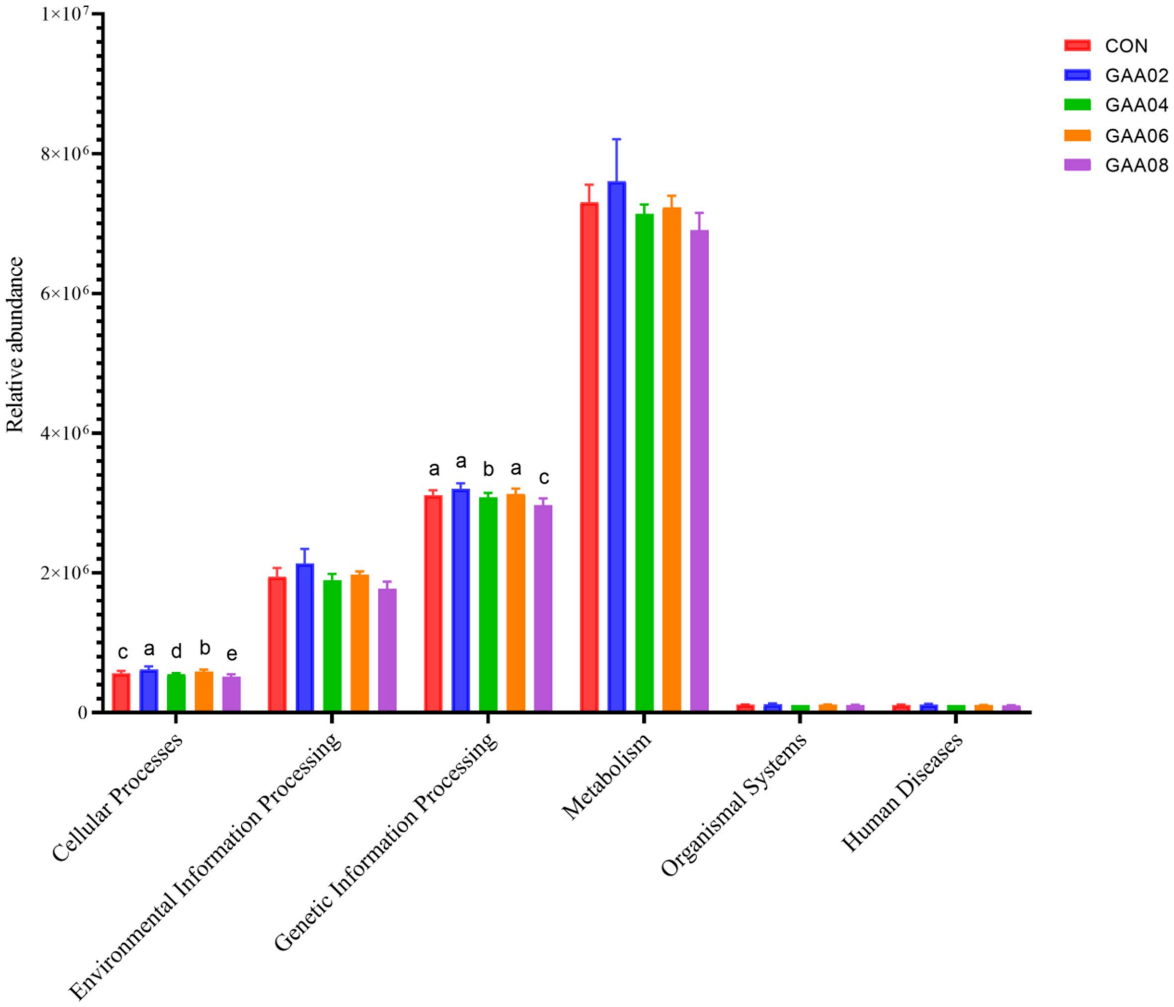
Primary metabolic pathway. Different letters in the same column indicate significant differences (*p* < 0.05). Different letters in the same column indicate significant differences (*p* < 0.05).

PICRUSt1 was used to predict gene function based on 16S amplicon sequencing results to evaluate the functional characteristics of rumen microbiota supplemented with different levels of GAA. A total of six primary metabolic pathway levels were obtained through KEGG database comparison (Figure 5), including cellular processes, environmental information processing, genetic information processing, human diseases, metabolism, and organismal systems. The results showed that the expression of cellular processes and genetic information processing in the GAA08 group was downregulated (*p* < 0.05).

**Figure 6:**
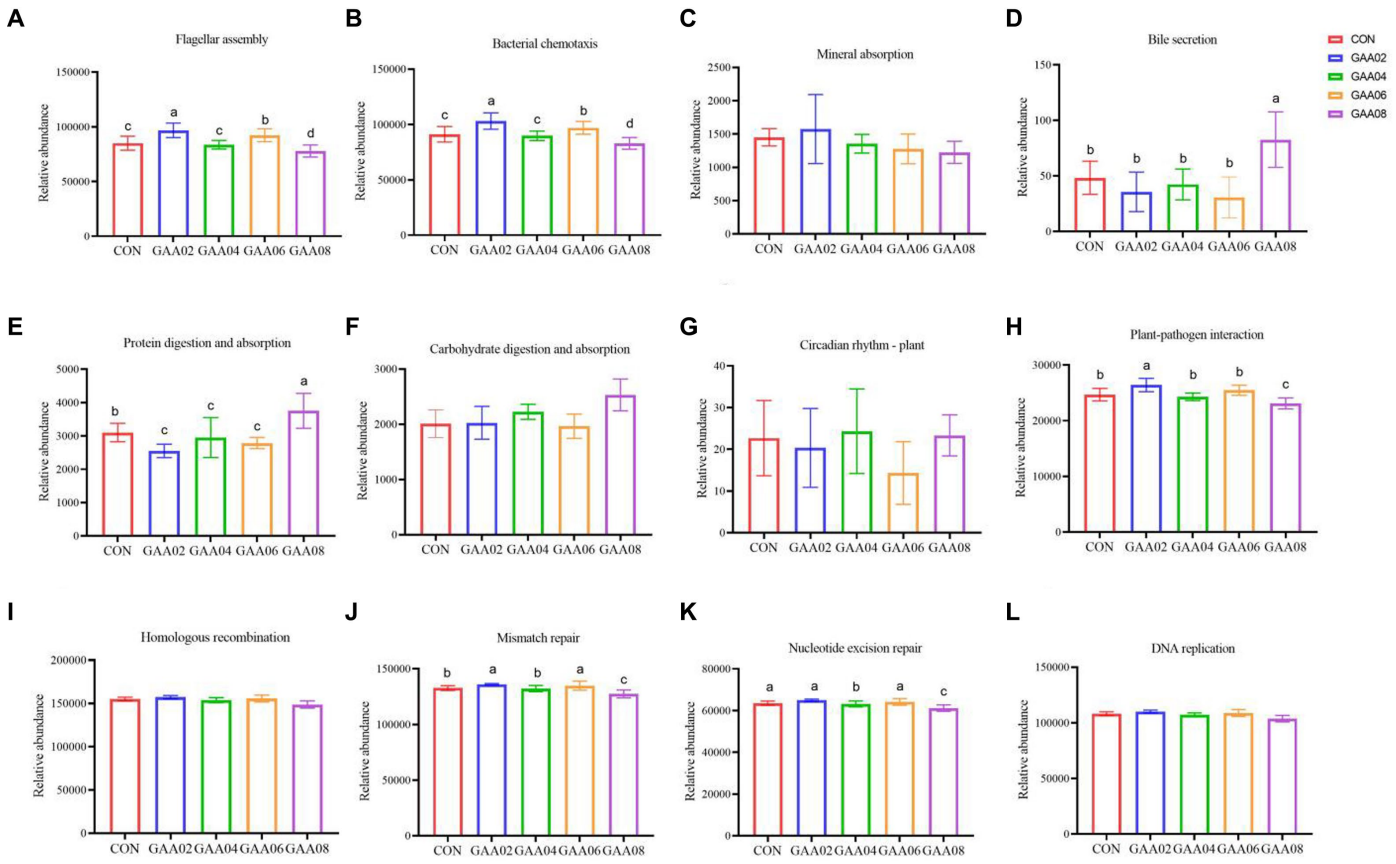
The tertiary metabolic pathway of cell motility **(A,B)**, tertiary metabolic pathways of the digestive system **(C–F)**, tertiary metabolic pathways of environmental adaptation **(G,H)**, and tertiary metabolic pathways of replication and repair **(I–L)**. Different letters in the same column indicate significant differences (*p* < 0.05).

Further analysis of the metabolic pathway is shown in Figure 6. In the GAA08 group, the expression of flagellar assembly, bacterial chemotaxis, plant pathogen interaction, mismatch repair, and nucleotide exclusion repair were all downregulated (*p* < 0.05). However, the expression of bile secretion and protein digestion and absorption in the GAA08 group was upregulated (*p* < 0.05).

## 4 Discussion

*In vitro* rumen-simulated fermentation techniques are commonly used to evaluate the extent of rumen microbial fermentation and the utilization of diets (Yejun et al., 2019; Grubješić et al., 2020; Choi et al., 2021). Rumen microorganisms ferment carbohydrates to produce rumen gasses (H_2_, CH_4_, CO_2_, etc.) and VFAs (Ju et al., 2023). Under the action of some microorganisms, these gasses also have a certain mutual transformation (Lv et al., 2022). Rumen gas production and gas composition reflect not only the digestion process of feed in the rumen but also the changes in rumen microorganisms and are usually affected by fermentation substrates and additives. It is obvious that substrate composition affects rumen fermentation kinetics. It has been shown that supplementing 0.4% GAA to a fermentation substrate with a ratio of concentrate to forage (C/F) of 7:3 can increase the total gas production after 24 h of fermentation (Ren et al., 2023), and a C/F of 5:5 can also promote nutrient digestion and rumen fermentation in Angus bulls (Li et al., 2020). Therefore, it is worth discussing the effect of GAA when the ratio of concentrate to forage is further reduced. In this experiment (with C/F 4:6), there was no significant difference in GP_48 h_ between the GAA groups and the CON group, but the gas production in the GAA groups was numerically lower (*p* > 0.05). In addition, the gas production rate is also an important indicator of rumen fermentation kinetics (Wu et al., 2022). In this experiment, the theoretical maximum gas production (B in Table 2) and gas production rate (C in Table 2) predicted by the model showed no significant differences between groups, and the gas proportions of H_2_, CH_4_, and CO_2_ did not change. Since rumen gas was mainly produced from carbohydrates in the diet, it is considered that GAA barely influenced carbohydrate digestion of the substrate with this C/F (4:6).

A relatively stable rumen environment is important for the colonization and function of rumen microorganisms; in general, the indicators examined include rumen pH, NH_3_-N and VFAs (Ma et al., 2022). Rumen pH is particularly important (Ma, 2021) because it is also the result of the combined effect of rumen VFA and NH_3_-N. The rumen pH fluctuates from 5.5 to 7.5, which is conducive to rumen microorganisms and to the normal function of ruminants (Calsamiglia et al., 2008). In this experiment, the pH of the rumen in each group fluctuated within the normal range of 6.09–6.13, indicating that GAA supplementation did not disrupt the balance of volatile acids and ammonia nitrogen and maintained the acid–base environment, which was consistent with the results of Wales et al. (2004).

The NH_3_-N concentration in the rumen is affected by the substrate’s degradable protein content and the activity of rumen microorganisms (Zain et al., 2023). The optimal NH_3_-N concentration for rumen microorganism growth is generally within the range of 20–50 mg/dL under *in vitro* conditions (Satter and Slyter, 1974). The results of this experiment showed that the NH_3_-N concentrations of all groups were similar and fluctuated within the normal range from 31.99 to 35.31 mg/dL, which means that microbial nitrogen sources were adequately supplied. Since GAA contains 35.86% nitrogen and does not affect the rumen ammonia nitrogen content but increases MCP, it is speculated that it promotes the conversion of NH_3_-N into MCP. The same result was reported in lambs (Ren et al., 2023). However, it may depend on more factors than the amount added. A decreased NH_3_-N concentration was also observed (Li et al., 2020; Liu et al., 2021). Supplementation with GAA (from 300 mg to 900 mg/kg) even affected the growth performance and nutrient digestion of lambs (Liu et al., 2021).

Carbohydrates are fermented in the rumen and produce VFAs, which can meet 70–80% of ruminant energy needs (Rey et al., 2014); therefore, carbohydrates are an important source of energy for ruminants. In addition, acetate and butyrate are important substances for milk fat synthesis, while propionate is an important precursor for glucose synthesis in gluconeogenesis, which is also mainly used for synthesizing body fat and lactose (Li et al., 2023). The proportion of VFA depends on the rumen fermentation pattern, especially C/F. More acetic acid and less propionic acid are produced when C/F is low. To distinguish rumen fermentation patterns, when A/P is greater than 3, it is considered an acetate-type fermentation. Otherwise, it is a propionate-type fermentation (Gong, 2020). The lower C/F in this experiment was achieved by acetate-type fermentation, but the concentration of propionate significantly increased with GAA supplementation. Therefore, the A/P showed a decreasing trend due to the increase in propionate. This result was consistent with the report of Liu et al. (2023). This meant that GAA supplementation has a regulatory effect on the rumen fermentation pattern to perform propionate-type fermentation. In fact, propionate-type fermentation has important physiological significance in finishing cattle, such as the lowest heat consumption, less methane production and the highest energy conversion efficiency. This means that beef cattle can obtain more energy for weight gain (Xie et al., 2021). The effect of GAA on the rumen fermentation pattern may be attributed to rumen microorganisms. The results of 16S in this experiment indicated that GAA supplementation increased the abundance of *Prevotella* and *Prevotellaceae_UCG-001*, which are involved in propionate metabolism in the rumen, and thus led to an increase in the concentration of propionate.

Branched chain amino acids (valine, leucine, and proline) undergo oxidative deamination or decarboxylation by rumen microorganisms (Ren and Zhao, 2008), which produce corresponding isomeric acids (butyrate, isobutyrate, isovalerate, and valerate) (Zhang et al., 2022). Among them, isovalerate is associated with fiber degradation and was reported to increase acetate, butyrate and TVFA in the rumen of Simmental cattle and reduce propionate, which leads to acetate-type fermentation (Liu et al., 2007). In this experiment, propionate significantly increased and isovalerate showed a significant downwards trend with increasing GAA addition. This means that isovaleric acid-related fiber degradation is weakened and propionic acid-related nonfiber carbohydrate degradation is enhanced. This was consistent with previous reports. However, GAA supplementation did not affect acetate and butyrate concentrations.

The rumen is a complex anaerobic fermentation system composed of various microorganisms with a high degree of microbial diversity (Mizrahi and Jami, 2018). These microorganisms are important in maintaining the stability of the rumen environment, increasing the nutrient digestion rate and maintaining animal productivity (Lin et al., 2018). Alpha diversity reflects the diversity within a specific ecosystem, the Shannon index is used to reflect microbial diversity, and the Chao index is an indicator of richness (Wei et al., 2017). Shabat et al. (2016) found that the diversity of rumen microorganisms decreased while the concentration of propionate increased. In this study, the same results were found. As the level of GAA addition increased, the Shannon index decreased, but the Chao index did not show significant changes. This suggested that supplementation with different GAA levels may partially affect the diversity of the rumen microbiota.

*Firmicutes* degrade dietary fiber to produce acetate and butyrate, while *Bacteroidota* mainly produces propionate through nonfibrous substance degradation. In this study, *Firmicutes* and *Bacteroidota* were also the dominant microbiota in the rumen, which was consistent with the results of other studies (Lee et al., 2011; Yang et al., 2020). However, the relative abundance of *Firmicutes* showed a trend of first increasing and then decreasing with GAA addition, while *Bacteroidota* showed a significant decrease at first and then increasing. The most obvious changes were observed within the GAA02 and GAA08 groups, and between them, they showed almost opposite results. This meant that the result may depend on the GAA level, and the 0.8% level may be a critical value. When the amount of GAA supplemented was 0.8%, *Bacteroidota* significantly increased, and the results of rumen volatile acids also indicated a significant increase in propionate concentration. This indicated that low levels of GAA may be beneficial for *Firmicutes* survival, while high levels of GAA are more suitable for *Bacteroidota* to degrade nonfibrous substances and produce more propionate.

Further research and analysis of the differences in genus-level communities revealed that *Rikenellaceae_RC9_gut_group* is the dominant genus in the rumen, which is the same as the reports of Liu et al. (2022) and Ahmad et al. (2022). Cox et al. (2014) found that *Rikenellaceae_RC9_gut_group* is a beneficial bacterium with intestinal protective functions that is mainly involved in the degradation of plant-based polysaccharides in the body, and its relative abundance is directly proportional to dietary fiber content (Fan et al., 2020). This indicates that GAA supplementation does not negatively impact dietary fiber degradation that can be used by beneficial dominant bacteria in the rumen. In this experiment, the addition of 0.6% GAA significantly increased *norank_f__F082* and *Papillibacter*. The *norank_f__F082* belongs to *Bacteroides* and mainly participates in nonstructural carbohydrate degradation. *Papillibacter* has been found to exist in the rumen in studies (Ma et al., 2020; Zhang et al., 2022), but the functions of *norank_f__F082* and *Papillibacter* have not been reported. The relative abundance of *Prevotella* and *Prevotellaceae_UCG-001* was significantly increased when 0.8% GAA was supplemented. *Prevotella* and *Prevotellaceae_UCG-001* belong to the phylum *Bacteroidei*, among which *Prevotella* has a strong ability to degrade nonstructural carbohydrates and proteins and can ferment sugars through the acrylic and succinic acid pathways to produce propionate (Flinth, 2008). By examining the correlation between rumen fermentation parameters and microbial communities, propionate concentration was positively correlated with the relative abundance of *Prevotellaceae_UCG-001*. The increase in the relative abundance of *Prevotella* often leads to an increase in propionate content (Poudel et al., 2019). This also explains the increasing rumen propionate concentration with increasing GAA addition levels from the perspective of the microbial genus level.

The rumen microbiota is a complex ecosystem closely related to the biology of the host, and species interactions are crucial for the stability of the community in healthy symbiotic microbiota (Clemente et al., 2012; Faust et al., 2012). A network diagram was drawn based on the correlation between species to reflect the interactions between species in the sample. Ramayo-Caldas studied the relationship between gut microbes in 518 pigs (60 days of age) through correlation network analysis and found that genus-level correlation network analysis revealed significant interactions between different genera of pig gut flora (Ramayo-Caldas et al., 2016). *Prevotella* and *Prevotellaceae_UCG-001* showed a positive correlation, while the two were negatively correlated with other bacterial genera. This was consistent with the results of the genus-level relative abundance analysis above, where the addition of 0.8% GAA significantly increased the relative abundance of *Prevotella* and *Prevotellaceae_UCG-001* while decreasing the relative abundance of *NK4A214_group* and *Christensenellaceae_R-7_group*. Through correlation network analysis, it was found that *NK4A214_group*, *Prevotella*, *Prevotellaceae_UCG-001* and *Christensenellaceae_R-7_group* are important genera in the rumen microbiota, which may partially affect the stability of the rumen microbiota.

Rumen microorganisms participate in body metabolism and provide nutrients to the host by decomposing dietary nutrients, and the abundance and function of microflora are closely related to the host (Vasta et al., 2019). Further analysis showed downregulated expression of flagellar assembly, bacterial chemotaxis and plant–pathogen interaction in the GAA08 group. Flagellar assembly and bacterial chemotaxis are important in the bacterial adhesion process (Wang, 2019). Zhang et al. (2022) showed that plant–pathogen interactions mainly involve pathogenic microorganisms plundering nutrients from host cells for survival and reproduction, while host plants use various defense strategies to inhibit pathogen growth. This indicates that high GAA levels reduce the cell motility ability of bacteria and the plant–pathogen interaction. In addition, Hamana et al. (2012) showed that replication-and repair-related genes may help the body restore the molecular structure of genes and reduce the damage caused by mismatching biological molecules. In this experiment, the expression of mismatch repair and nucleotide excision repair in the GAA08 group was downregulated, indicating that supplementing high levels of GAA may reduce the body’s ability to replicate and repair biomolecules. The relative abundance of bile secretion and protein digestion and absorption in the GAA08 group was significantly higher than that in the other groups. Based on the above results of MCP concentration and relative abundance of bacteria, the addition of 0.8% GAA promotes nutrient utilization, such as lipids and proteins, by rumen microorganisms, leading to an increase in these genes.

## 5 Conclusion

The results showed that when GAA was supplemented, the concentrations of MCP and propionate in the rumen could be significantly increased, but isovalerate was decreased. In addition, the relative abundances of Bacteroidota, *Prevotella* and *Prevotellaceae_UCG-001* could be significantly enhanced. The metabolic pathways related to bile secretion, protein digestion and absorption were increased. In conclusion, supplementation with 0.8% GAA can enhance *in vitro* rumen fermentation parameters and increase the relative abundance of bacteria related to nonfiber substance degradation, which would enable beef cattle to obtain more energy.

## Data availability statement

The datasets presented in this study can be found in online repositories. The names of the repository/repositories and accession number(s) can be found below: NCBI - PRJNA1011887.

## Ethics statement

The animal studies were approved by Laboratory Animal Ethics Committee of Inner Mongolia Minzu University. The studies were conducted in accordance with the local legislation and institutional requirements. Written informed consent was obtained from the owners for the participation of their animals in this study.

## Author contributions

CD: Conceptualization, Data curation, Formal analysis, Writing – original draft. MW: Funding acquisition, Project administration, Resources, Writing – review & editing. JJ: Conceptualization, Writing – review & editing. LD: Methodology, Writing – review & editing. RZ: Investigation, Writing – review & editing. MX: Investigation, Writing – review & editing. YZ: Visualization, Writing – review & editing. HB: Visualization, Writing – review & editing. MB: Supervision, Writing – review & editing.
